# Sites of chromosomal instability in the context of nuclear architecture and function

**DOI:** 10.1007/s00018-020-03698-2

**Published:** 2020-11-21

**Authors:** Constanze Pentzold, Miriam Kokal, Stefan Pentzold, Anja Weise

**Affiliations:** 1 Institute of Human Genetics, University Hospital, Friedrich Schiller University Jena, 07747 Jena, Germany; 2 Research Center Lobeda, Jena University Hospital, 07747 Jena, Germany

**Keywords:** Cancer, Cell cycle, Chromatin organization, Chromosome condensation, Chromosome territory, Replication-transcription conflicts

## Abstract

Chromosomal fragile sites are described as areas within the tightly packed mitotic chromatin that appear as breaks or gaps mostly tracing back to a loosened structure and not a real nicked break within the DNA molecule. Most facts about fragile sites result from studies in mitotic cells, mainly during metaphase and mainly in lymphocytes. Here, we synthesize facts about the genomic regions that are prone to form gaps and breaks on metaphase chromosomes in the context of interphase. We conclude that nuclear architecture shapes the activity profile of the cell, i.e. replication timing and transcriptional activity, thereby influencing genomic integrity during interphase with the potential to cause fragility in mitosis. We further propose fragile sites as examples of regions specifically positioned in the interphase nucleus with putative anchoring points at the nuclear lamina to enable a tightly regulated replication–transcription profile and diverse signalling functions in the cell. Consequently, fragility starts before the actual display as chromosomal breakage in metaphase to balance the initial contradiction of cellular overgrowth or malfunctioning and maintaining diversity in molecular evolution.

## Chromosomal fragile sites within the replicative landscape of the nucleus

### The dilemma of cellular division

Developing organisms rely on cell division (mitotic cells) to distribute their genetic material equally and ideally errorless to subsequent daughter cells. During differentiation most cells do not continue to divide (postmitotic cells) and transition into senescence while the whole organism is ageing. For a long time, this course into cellular expiration by telomere shortening was thought to counteract cellular division progress into malignant overgrowth [1]. However, the constraint of mutational accumulation as a positive effect faces the secretion of tissue disrupting substances by senescent cells as negative effect [2, 3]. Concomitantly, cellular division continues even in mature organisms to some extent specifically to function for tissue maintenance and regeneration.

Every cell is exposed to up to thousands of single lesions from exogenous and endogenous sources to mostly challenge the integrity of the genetic material within one generation when affecting somatic cells [4, 5]. However, when germ cells are affected, mutations can be passed on to subsequent generations, thereby leading to a number of about 70 de novo mutations per diploid human genome per generation with a rate of 0.35 deleterious amino acid mutations per diploid genome [6] and might predispose to cancer development [7]. Hence, mutations accumulate in the course of cellular division that will be positive, negative or neutral for the affected organism.

### Impaired replication causes chromosomal fragility

One of the major drivers of endogenous DNA damage is replication, i.e. the accurate copying of the genetic material into two identical copies, each kept available for the spreading to two daughter cells during cell division. Hindered and slowed replicative processes in interphase can lead to stretches of under-replicated DNA where doubling of the genetic material is not finished on time before the onset of mitosis [8]. Furthermore, lack of replication origins [9], transcription–replication encounters [10, 11], steric hindrance as well as fork collapse [12] may hinder timely termination of replication additionally in interphase [13]. Such replication disturbances can lead to the expression of so-called chromosomal fragile sites (FSs) in metaphase [14]. These are cytogenetically appearing gaps or breaks on the most condensed form of the genetic material, on metaphase chromosomes [15, 16]. Most likely FSs are not representing real “broken”, meaning nicked, structures but rather decondensed ‘intercalary’ chromatin as also one of the first descriptions on chromosomal fragility refers to [17]. A recent study even suggests a failure of correct condensin loading to FSs before the onset of mitosis what leads to insufficient chromatin compaction resembling characteristic mitotic FS lesions [18].

### Grouping chromosomal fragile sites

Traditionally, fragile regions have been subdivided into ‘common fragile sites’ (CFSs) and ‘rare fragile sites’ considering the frequency of appearance of these sites in a normal population [16]. During the last years, scientists proceeded to subgroup only into CFSs and ‘early replicating fragile sites’ (ERFSs) [23] while omitting ‘rare fragile sites’ due to their direct linkage to specific sequence features [e.g. CCG triplet repeats in the case of FRAX (‘Fragile X Syndrome’ OMIM #300624)] [24] instead of referring to the formerly used mere cytogenetic definition. In comparison to CFSs, ERFSs are replicating early in S-phase, have relatively higher chromatin accessibility, a high density of replication origins, as well as a high G-C content [23]. At ERFSs, the integrity of replication is challenged by an increase in initiating events resulting in either replication–transcription conflicts or depletion of nucleotide pools [23, 25]. Both scenarios slow down replication leading to increased fragility [25, 26]. In contrast, deficiency of replication initiation events causes a reduced replication progression at CFSs [9, 23]. However, the fragility at both sites is increased by ATR inhibition, oncogenic stress and deficiencies in homologous recombination [16, 27, 28].

### How the nuclear interphase architecture might inform about chromosomal fragility

The above mentioned understanding of fragility is not any more based on the attempt to define parallel mechanisms to explain every breakage event, but rather use the term ‘fragile site’ as a converging definition of multifactorial aspects resulting in the same cytogenetic manifestation. What we see is most likely the consequence of earlier events and not the cause of further downstream events, such as breakage and genomic rearrangements. The defined mitotic instability of FSs raises questions about events in preceding interphases leading to a later uncondensed chromatin structure. While some genetic regions are known to replicate early in S-phase of the cell cycle, others are usually late replicating. This temporal difference is also reflected in the spatial organization of the genome during interphase. Whereas the nuclear centre usually harbours early replicating sites, the nuclear periphery is mostly composed of late replicating areas of the genome [19]. Since each chromosome occupies a certain space within the nucleus, known as chromosomal territory [20, 21], and since each chromosome itself possesses early as well as late replicating regions, it is evident that there is a higher order structure and organization even within the interphase nucleus without vigorous condensation of chromosomes [21, 22].

Are FSs genomic regions that purely represent the existence of large and difficult-to-replicate units with a low number of activatable origins leaving behind large stretches of DNA that cannot be replicated timely within one cell cycle [9, 29–33]? Or, alternatively, are FSs somehow actively hindered from replicating early, which would prevent termination of replication before the onset of mitosis?

## Chromosomal fragility—mitotic instability tracing back to nuclear positioning during interphase

### The role of transcription in replicatively challenged genomic regions

The before-mentioned replication challenges seem to cause delayed chromosomal condensation in later cell division cycle stages. Transcription may further delay condensation of late replicating regions for mitotic chromosomal separation. The coincidence of replication and transcription causes those two acting multiprotein machineries to encounter in the same genomic region thereby suppressing replication initiation [33] or even producing DNA lesions, structural variants and under-replicated DNA stretches that can form FSs [11, 34–36]. Interestingly, replication-transcription conflicts do not activate the cell cycle checkpoint completely what might resolve encounter intermediates before mitotic entry by continued replication [37]. However, acetylation, the histone mark that is connected with chromatin accessibility, has been reported to be present in a hypoacetylated state at FSs [38]. Hypoacetylation leads to a relatively more compact chromatin structure and seems to hinder gene replication, expression and repair at these sites [39]. Besides hypoacetylation, gene repression is usually characterized by histone marks, such as H3K9me2 and H3K9me3, which are enriched beneath the nuclear lamina, which is a structural reticulation underlying the inner membrane of the nucleus. Chromatin that is linked to the nuclear lamina is organized in so-called lamina-associated domains (LADs) [40, 41]. These LADs, especially the outer 200 kb of LADs are characterized by repressive H3K9 methylation, most likely to prevent spreading of active chromatin into heterochromatic regions [42], thereby maintaining cell type-specific gene expression profiles. So far it is not known whether the tethering of genetic regions to the lamina follows the chromatin marks that are footprints of their transcriptional activity [43, 44] or whether the tethering itself leads to transcriptional repression as well as late replication [40, 45]. By now there is evidence for both theories [40, 44]. A recent study points on the direct regulation of the replication profile by a certain threshold of transcription, i.e. high expression levels promote earlier replication to prevent incomplete replication before the onset of mitosis [46]. Consequently, the nuclear lamina is not only an important player of the structural nuclear architecture and inner compartmentalization of the nucleus but also of functional gene regulation [47]. Interestingly, FSs that were previously thought to simply represent the mitotic result of DNA damage and insufficient repair in the preceding interphase are found to have overlapping characteristics with LADs [40–42]: spanning large regions from 100 kb to 10 Mb with a low gene density, late replicating in interphase, lowly transcribed, comprising large genes with at least 500 kb size, high A/T content and repressive chromatin marks [10, 38, 48–53].

### The putative link between the nuclear periphery and FS manifestation

The so-far mainly cited reasons for chromosomal fragility summarize in a set of large genes and replication units that are late replicating and concomitantly transcribed in interphase [53]. However, the processes of replication and transcription seem to be timely and spatially separated within the nucleus. The replicative spatial organization is closely linked to transcriptional activity whereby highly transcribed regions (euchromatin) are found in the nuclear centre and lowly transcribed or repressed genes (heterochromatin), respectively, at the nuclear periphery [54]. Thereby, peripheral areas within the nucleus are determined by gene-poor, mid-to-late replicating and lowly transcribed genomic regions in close vicinity to the nuclear lamina [20, 55].

Thus, genomic regions later manifested as FSs should be located in the nuclear periphery during interphase concerning their replication profile, but closer to the nuclear interior concerning their transcriptional profile. A peripheral location favours the idea of possible nuclear membrane or cytoskeletal anchor points that prevent certain genomic areas from early replication, hamper them from being transcribed that may leave behind uncondensed chromatin in mitosis when they continue to do anyway. A low transcription level is sufficient to cause later fragility [46, 53] indicating that a low threshold may already cause imbalance in mitosis. However, a higher transcription level might favour earlier replication to avoid mitotic instability [46]. Given that FS have been linked to neurological disorders, it is tempting to assume a signalling function of FSs to constrain expression of neurologically active gene products to better control expression as in these vulnerable areas protein overexpression might have severe outcomes [48, 56, 57]. This idea is supported by the fact that FSs are tissue specifically expressed [9, 50] what mirrors tissue-specific replication timing and expression program of each cell type [58, 59].

Despite it is object of recent discussion, the possible link between lamina anchorage in interphase and chromosomal breakage in mitosis has not been addressed in detail yet [40, 60]. This raises the question how nuclear organization is maintained through each cell cycle and how non-genetic information is passed on during cell division to maintain cell type-specific replication, transcription and thereby developmental profile. Possible answers may involve the role of interphase nuclear architecture for chromosomal stability.

## The nuclear architecture is a main driver of chromosomal organization, regulation and stability

### The relation of FSs and nuclear pore complexes

The nuclear material has a cell type-specific organization, i.e. the relation of chromosomes to each other and to the nuclear periphery is specific to each cell type [21, 55]. This information is maintained through mitosis. The double membrane layered nuclear envelope is interspersed by nuclear pore complexes (NPCs) that enable interchange of substances between the cyto- and nucleoplasm. These NPCs are often associated with euchromatic regions thereby interrupting the peripheral heterochromatic area within the nucleus [55, 61].

Partially it has been described that FSs are located in the nuclear periphery—visualized by the localization of FANCD2 [62] what indicates FSs [53]. These findings suggest a late replicated, but generally repressed state of the chromatin in the course of interphase; however, FSs are transcribed at least to some extent. This prompts the question whether these peripheral FSs are located close to the nuclear lamina or might be associated to NPCs? If they were localized at NPCs, this would explain their transcriptional activity and late replication might refer to long-lasting rather than late onset replication. Long lasting replication is supported by the fact that even early mitotic replication exists, mainly at difficult-to-replicate and otherwise lesion prone regions, such as FSs [63, 64]. However, this would implicate that FSs were not located at the nuclear periphery because FSs are late replicating themselves and by chance transcribed, but rather transcribed on purpose. Hence, FSs should be processed in the periphery with close connection to protein exchange locations, such as NPCs as well as repair factories [65–67]. If the localization of FSs was within the periphery but distant from NPCs, meaning within LADs, this would argue in favour of the hypothesis that late onset replication is meaningful in FS expression. However, a certain level of concomitant transcription—that is not designated for peripheral heterochromatic areas—might be the leading fact towards later fragility as two processes coincide that should be separated in the interphase nucleus. Thereby specific anchor points in the nuclear lamina for these genomic regions are conceivable as exemplified consecutively.

### Anchor points at the nuclear lamina as potential sources of fragility

In embryonic cells of fruit flies it was shown that lamina interacting regions are characterized by a certain degree of AT-richness, transcriptional repression with according histone modifications, late replication and long intergenic distance [54]. Similar features have been described for FSs [17, 31, 38, 49, 68] and the intergenic spacing matches the recently described withstanding of FSs in intron size reduction in an avian species in the course of general genome size reduction of these vertebrates [53]. Interestingly, certain anchor points of lamina proteins for DNA have already been found to contribute to genome instability. For example, CTCF-sites, i.e. DNA binding sites for the transcriptional zinc-finger repressor CTCF (‘CCCTC-binding factor’), have major functions in structural chromatin and transcriptional regulation mainly as so-called ‘topological associated domains’ (TADs) [21, 69]. CTCF-sites can directly anchor DNA to the nuclear lamina, thereby arranging chromatic loop structures to define eu- and heterochromatic regions. Interestingly, within these loop anchor points a higher number of structural variants is found and FSs seem to overlap with these anchorage sites [60, 70]. With this looping, genomic regions are either allocated to central active or peripheral repressed chromatin with CTCF sites constituting a domain boundary [44] and NPCs are involved in the setting of chromatin looping [61]. Thereby chromatin anchors and loops seem to regulate transcriptional activity to set cell type-specific gene expression profiles. This is in accordance with tissue-specific organization of chromatin looping and transcriptional regulation where the basal chromosome architecture is cumulatively altered at hundreds of sites suggesting that lamina-genome interactions are widely involved in the control of gene expression [44, 47, 71]. Several genes have been used in repositioning assays and monitored cell type specifically to identify the dependence of nuclear positioning on their expression profile and developmental status [45, 72, 73]. Consequently, the cellular differentiation status regulates the movement of genomic regions from the nuclear periphery to the interior to change their expressional state and TADs appear as dynamic structures in the course of differentiation [41, 74].

**Fig. 1:**
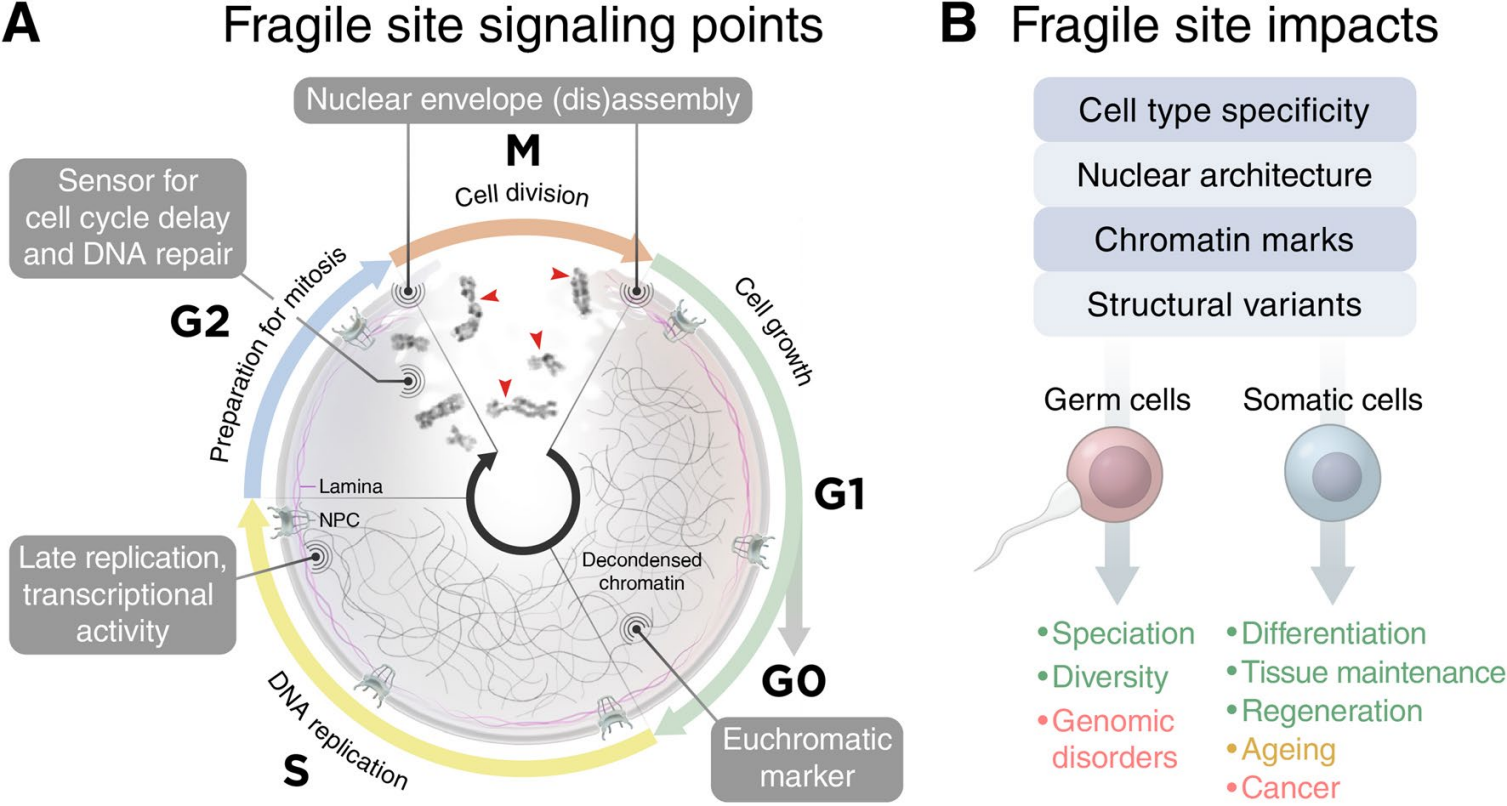
The genetic material is packed within the nucleus, comprising of a nuclear envelope, the underlying nuclear lamina including trans-membrane proteins, such as NPCs and chromatin, which can be subdivided in two regulatory compartments. These two compartments usually represent an early replicating, gene-rich, transcriptionally active (euchromatic) central region and a late replicating, gene-poor, more transcriptionally repressed (heterochromatic) peripheral region, respectively. Chromosomal fragile sites (FSs) exert important cellular functions in the course of cell division cycle and development by combining characteristics of both compartments. **a** FSs act as sensors for cell cycle delay and DNA repair, nuclear envelope modelling as well as a euchromatic marker in a heterochromatic surrounding to prevent chromosomal instability. **b** FSs accomplish cell type-specific activity profiles, stabilize the nuclear architecture and are regions of structural variants to balance beneficial and detrimental effects on an organismal and evolutionary scale. NPC- nuclear pore complex. G0, G1, G2, S, M—cell cycle phases (*G* gap, *S* synthesis, *M* mitosis)

Another example for chromatin-lamina-interaction is the lamin B receptor which is the direct link between lamins and chromatin [75]. It was shown that specific sequences are associated with interacting regions, such as extended GAGA motifs, that when used in repositioning assays will target chromatin to the nuclear lamina; in contrast, regions that were originally not positioned in the nuclear periphery, replaced their ectopic genetic regions also to the interior nucleoplasm [40]. Furthermore, these repositioned sites are thought to be changed in their expression activity according to their new genetic surrounding [40, 45]. This is a hypothesis based on the fact that the insertion of a reporter gene results in a lower expression within an LAD than in the nuclear centre and that this process is closely linked to histone deacetylation which is a mark of repressed chromatin and FSs [38, 40]. Vice versa LAD-sequences inserted elsewhere in the genome will be tethered to the nuclear periphery and repressed. However, there are contradictory results from a similar approach where repositioning of a former transcriptionally active and nuclear interior locus into the nuclear periphery maintained its transcriptional profile after one cell cycle [76]. Nevertheless, these observations are not mutually exclusive. Differentiation and the associated rearrangement status of a cell, localization in connection to NPCs as well as certain sequence features can result in a defined transcription pattern also at the nuclear periphery [47]. Therefore, we propose that any genomic region that is tethered to the nuclear periphery and thereby defined by its traits, such as late replication, low transcription activity, gene poverty, larger gene and intergenic size, without a certain sequence specificity can result in a mitotic FS (Fig. 1).

### Evidence for fragility due to nuclear matrix attachment

Recently published data suggests that DNA regions tightly connected to chromatin loop anchors or to the nuclear matrix (NM) are involved in chromosomal fragility in a cell type-specific manner [60, 77]. The NM is a substructure containing the nuclear lamina including NPCs, the nucleolus, and the intranuclear fibrous network [78] and plays a major role in the structure and function of the nucleus [21]. It was revealed that the mouse genomic region Fra14A2/*Fhit*, an ortholog to the human FRA3B, is largely associated with the NM in naïve B lymphocytes where FRA3B is a highly expressed CFS. The same region is only partially anchored to the NM in primary hepatocytes and postmitotic neurons which are not expressing this CFS. Furthermore, global analyses of structural DNA-NM interactions in naïve B lymphocytes displayed that most of the genome has no tight interaction, emphasizing that the fragility of Fra14A2 might be related to the association with the NM [77]. The embedding of DNA to the NM might result in a scarcity of typical origins of replication and replicons [9]. This lack of topological DNA features can lead to stress and therefore to genomic instability resulting in chromosomal fragility [79].

### Nuclear lamina-associated FSs have a balancer role in cell development and evolution

The question remains what establishes first during development: inactive genomic regions marked by specific repressive chromatin marks will be read out to be tethered to the nuclear periphery thereby creating two nuclear regions leaving space in the centre for protein accumulation and transcription [41], and all repressed chromatin is in the periphery tightly packed for repression enhancement by steric hindrance of protein entrance. Alternatively, all genomic regions tethered by nuclear lamina proteins will be shuttled to the periphery [44], thereby creating regional specific chromatin marks and subsequently establishing a repressive state of the chromatin. Whatever processes lead to cell type-specific expression profiles, their first establishment successively results in a dynamic rearrangement during differentiation. Nuclear material usually demonstrates slow movements within the nucleus and stable positioning within one cell cycle [80]. However, damaged telomeres and elsewise damaged DNA areas can move through the nucleus for relocation to repair centres and transcriptionally active chromatin will also move within the nucleus [67]. Chromatin association to the nuclear periphery is changed upon transcription and thereby movements are thought to be limited by active RNA polymerase II creating so-called transcription factories for facilitated protein progression [81, 82]. Since chromatin movement is constrained within a cell cycle but should be favoured during cellular differentiation there seems a discrepancy between short term cell survival and long term differentiation and evolution. Therefore we hypothesize a balancer role of nuclear lamina-associated FSs that are accompanied by certain activity profiles. This limits the amount of active *versus* repressed chromatin and DNA damage on a cellular *versus* evolutionary level.

## Conclusion, main messages and future perspectives

The cytogenetic appearance of FSs as gaps and breaks has mainly been assigned to under-replicated DNA stretches that might be resolved by diverse repair pathways before the onset of mitosis to prevent chromosomal instability. Specifically in cancer cells, chromosome integrity and recurrent copy number alterations are discussed with involvement of FSs [13, 36]. It is still debated how cell cycle is delayed to ensure a certain amount of repair, replication completion and sufficient chromatin compaction on the one hand [37]; but not to stop cell cycle progression leading to growth arrest and eventually cell death on the other hand. It has also been suggested that late replication in large genes with high intronic content constitutes the lowest chance of genetic information loss when escaping the check point [83]. Like this, specific setting of FSs in large genes would overall minimize genomic instability.

The time of manifestation and visibility of chromosomal fragility is not necessarily the time of occurrence of an actual DNA damage. By focusing on the factors contributing to imbalances at the time of their action, but not at the time of visible consequences, it seems that even primarily assessed detrimental lesions can have beneficial functions on a cellular and evolutionary level. Therefore, in addition to consider FSs as harmful chromosomal lesions, they also have a certain function for chromosomal stability as sites of uncondensed chromatin, similarly to constrictions, such as centromeres or nucleolar organizing regions at the short arms of acrocentric human chromosomes.

By further contemplating the conservation of FSs not only within the mammalian lineage [84–87] but also in avian species [53, 88] and even the occurrence of somewhat similar sites in insects [89], yeast [90] and plants [91, 92], it is tempting to speculate on the function of these sites. We suggest a balancer and memory function of FSs in the context of the incompatibility of cell division and cellular senescence to keep an equilibrium between the chance of self-renewal and regeneration as well as cancerogenic overgrowth and ageing, respectively.

We, thus, propose FSs as** signalling points** (Fig. 1a) combining:

(I) late and/or long-lasting replication as a sensor for cell cycle delay preventing genomic instability in subsequent cell divisions,
(II) transcriptional activity in a surrounding of delayed replication for clashes leading to DNA instability as sensors for DNA repair,
(III) transcriptional activity in a surrounding of delayed replication to antedate replication timing to prevent genome instability,
(IV) transcriptional activity accompanied by nucleosome exclusion as a marker of euchromatic regions associated with a decondensed chromatin structure and
(V) anchoring to the nuclear lamina as a sensor for nuclear envelope disassembly and reassembly before and after mitosis on a cellular level.

Additionally, FSs merge the** functions** (Fig. 1b) of:

(I) recognition sites for the establishment of cell type-specific transcriptional profiles,
(II) balancing gene expression in a surrounding of tightly regulated expression profiles and areas with a low threshold towards changing expression—specifically in the brain,
(III) recognition sites for the determination and maintenance of the nuclear architecture after cell division,
(IV) platforms for non-genetic chromatin marks as reusable evolutionary hot spots in subsequent generations and
(V) hot spots for structural variants as a source of diversity on an organismal as well as evolutionary level.

Therefore, future studies should address in more detail functional and beneficial consequences of FS expression with the focus on their caretaker functions. It should be asked whether every site that is physically anchored to the periphery can indeed result in a mitotic FS and whether FSs per se are located at the nuclear periphery in the course of cellular differentiation.
